# Real-world surgical outcomes of segmentectomy vs. lobectomy in older adults with small stage IA NSCLC: long-term cancer-specific and competing mortality in an overlap-weighted SEER cohort

**DOI:** 10.3389/fmed.2026.1976284

**Published:** 2026-10-27

**Authors:** Jiasi Zhou, Shengying Zhang, Lili Liang, Yifeng Ren, Haichuan Fang

**Affiliations:** 1 Department of Pulmonary and Critical Care Medicine, Ningbo Hospital of Integrated Traditional Chinese and Western Medicine, Ningbo, Zhejiang, China; 2 Department of Rheumatology and Immunology, The People’s Hospital of Yuhuan, Yuhuan, Zhejiang, China; 3 Department of Emergency Medicine, The Affiliated People’s Hospital of Ningbo University, Ningbo, Zhejiang, China

**Keywords:** competing risks, lobectomy, non-small cell lung cancer, older adults, overlap weighting, real-world evidence, SEER, segmentectomy

## Abstract

**Background:**

Segmentectomy preserves more pulmonary parenchyma than lobectomy, but whether this translates into lower competing mortality in older adults is uncertain.

**Objective:**

To compare lung cancer-specific and other-cause mortality after segmentectomy vs. lobectomy in older adults with small stage IA non-small cell lung cancer (NSCLC) treated in routine US practice.

**Methods:**

We used Surveillance, Epidemiology, and End Results data to identify adults aged ≥70 years with a first primary, 1–20 mm, stage IA NSCLC treated with segmentectomy or lobectomy. The primary 5-year cohort included diagnoses from 2010 to 2018 and the secondary 3-year cohort diagnoses through 2020. Propensity-score overlap weighting, competing-risk methods, cause-specific Cox models, Fine-Gray models, and 1,000-replicate bootstrap inference were used. Additional sensitivity analyses quantified unmeasured-confounding robustness with *E*-values and repeated the primary analysis after restricting to patients with ≥1, ≥6, or ≥10 regional nodes examined, with propensity scores re-estimated within each restricted cohort.

**Results:**

The primary cohort included 4,072 patients (430 segmentectomy; 3,642 lobectomy). Five-year lung cancer-specific mortality was 15.23% after segmentectomy and 11.15% after lobectomy (RD, +4.08 deaths per 100 patients; 95% percentile bootstrap CI, +0.63 to +7.31); the cause-specific HR was 1.31 (95% CI, 1.05–1.63). Other-cause mortality was similar. The primary *E*-value was 1.71 for the point estimate and 1.23 for the confidence-limit estimate. With increasingly stringent nodal-evaluation restrictions, the lung cancer-specific association attenuated and became less precise; for ≥6 examined nodes, the 5-year RD was +4.18 per 100 (95% CI, −1.04 to +9.58), and for ≥10 nodes it was +1.88 per 100 (95% CI, −4.58 to +8.59).

**Conclusions:**

In routine practice, segmentectomy was associated with higher 5-year lung cancer-specific mortality in the primary analysis, but attenuation under more extensive nodal evaluation and modest *E*-values indicate that patient selection and procedural quality may materially contribute. These findings should not be interpreted as evidence of an intrinsic causal disadvantage of segmentectomy in appropriately selected patients.

## 1 Introduction

Lung cancer remains the leading cause of cancer death worldwide, and most cases are non-small cell lung cancer (NSCLC) (1). In the United States, lung cancer remains the leading cause of cancer death, with an estimated 125,070 deaths in 2024 (2). Screening with low-dose computed tomography has increased the detection of small, potentially curable lung cancers (3, 4). As more patients are diagnosed with early-stage disease, the surgical question has changed. The issue is no longer only whether a tumor should be removed, but how much lung should be removed while maintaining reliable cancer control (5).

For many years, lobectomy was the standard operation for stage I NSCLC because the Lung Cancer Study Group trial reported more local recurrence after limited resection (6). Modern trials have changed this view. JCOG0802/WJOG4607L showed better overall survival with segmentectomy than lobectomy in selected patients with peripheral NSCLC ≤2 cm, although local relapse was more common after segmentectomy (7). CALGB/Alliance 140503 showed that sublobar resection was noninferior to lobectomy for disease-free survival when peripheral tumors ≤2 cm were carefully staged and lymph nodes were confirmed negative (8). JCOG1211 also showed excellent outcomes after segmentectomy for ground-glass-dominant tumors (9). These studies established segmentectomy as an appropriate operation for selected early-stage tumors, provided that patient selection, margins, and nodal assessment are adequate (10).

The choice may be different in older adults. Segmentectomy usually preserves more postoperative pulmonary function than lobectomy (11). This is attractive in patients with reduced cardiopulmonary reserve, and segmentectomy has become more common in contemporary practice (12). However, better pulmonary function does not automatically mean longer survival. An older patient can die from cardiovascular, pulmonary, or other non-cancer causes, while a small difference in local cancer control may not become apparent for several years. For this reason, overall survival alone may not show the full balance between preserving lung function and maintaining long-term cancer control.

Previous studies in older patients have not settled this issue. SEER analyses in patients aged ≥70 or ≥75 years have reported mixed results, with some studies supporting segmentectomy in selected patients and others favoring lobectomy or showing that outcome depends on tumor size and surgical details (13–15). A meta-analysis of elderly patients found broadly similar survival after segmentectomy and lobectomy, but most included evidence was observational (16). A recent SEER study of stage IA lung adenocarcinoma ≤20 mm also found no significant mortality difference after propensity-score matching (17). At the same time, *post hoc* analyses of JCOG0802 have raised a different question. In pure-solid tumors and in tumors with invasive pathologic features, segmentectomy remained associated with more locoregional relapse, while the 2026 analysis reported fewer deaths from other causes after segmentectomy (18, 19). This suggests that any overall-survival benefit from segmentectomy may partly come from reducing competing mortality rather than from better cancer control.

The present study was designed to answer two related questions in routine clinical practice. First, among adults aged ≥70 years with a small stage IA NSCLC, is segmentectomy associated with fewer deaths from causes other than lung cancer, as would be expected if preservation of lung tissue produces a survival benefit? Second, is lung cancer-specific mortality different after segmentectomy vs. lobectomy, and does that difference become more apparent with longer follow-up? We used SEER data, propensity-score overlap weighting, competing-risk methods, and absolute risk differences at fixed time points to answer these questions. The primary analysis focused on 5-year outcomes, and a secondary 3-year cohort was used to assess whether the same pattern was present in more recent practice.

## 2 Materials and methods

### 2.1 Study design and data source

We conducted a retrospective population-based cohort study using the Incidence—SEER Research Data, 17 Registries, November 2025 Submission. These data include cancers diagnosed from 2000 through 2023 and cover approximately 26.5% of the US population based on the 2020 census (20). SEER records demographic characteristics, tumor features, first-course treatment, survival, and cause of death. The study was reported according to the STROBE statement (21).

The source file was a SEER case listing of malignant respiratory and intrathoracic tumors. All eligibility criteria were applied programmatically before analysis. SEER research data are deidentified. Therefore, individual consent was not applicable, and institutional review board review was not required under the policies that apply to secondary analysis of deidentified public-use data. The study complied with the SEER Research Data Use Agreement.

### 2.2 Patient population and analysis windows

The source export contained 1,282,136 records. We first limited the data to diagnoses from 2010 to 2023 (*n* = 760,328), primary lung malignancy (*n* = 690,309), age ≥70 years (*n* = 378,481), and NSCLC histology (*n* = 285,585). We then retained patients with one primary cancer or the first of multiple primary cancers (SEER sequence 00 or 01), leaving 190,218 records. Tumor size was limited to 1–20 mm using the SEER Tumor Size Over Time Recode (*n* = 34,158). We next required era-specific stage IA disease (*n* = 18,425), excluded one record with a recorded positive regional node, and retained only segmentectomy or lobectomy. The final 2010–2023 utilization cohort included 7,275 patients (Figure 1).

**Figure 1:**
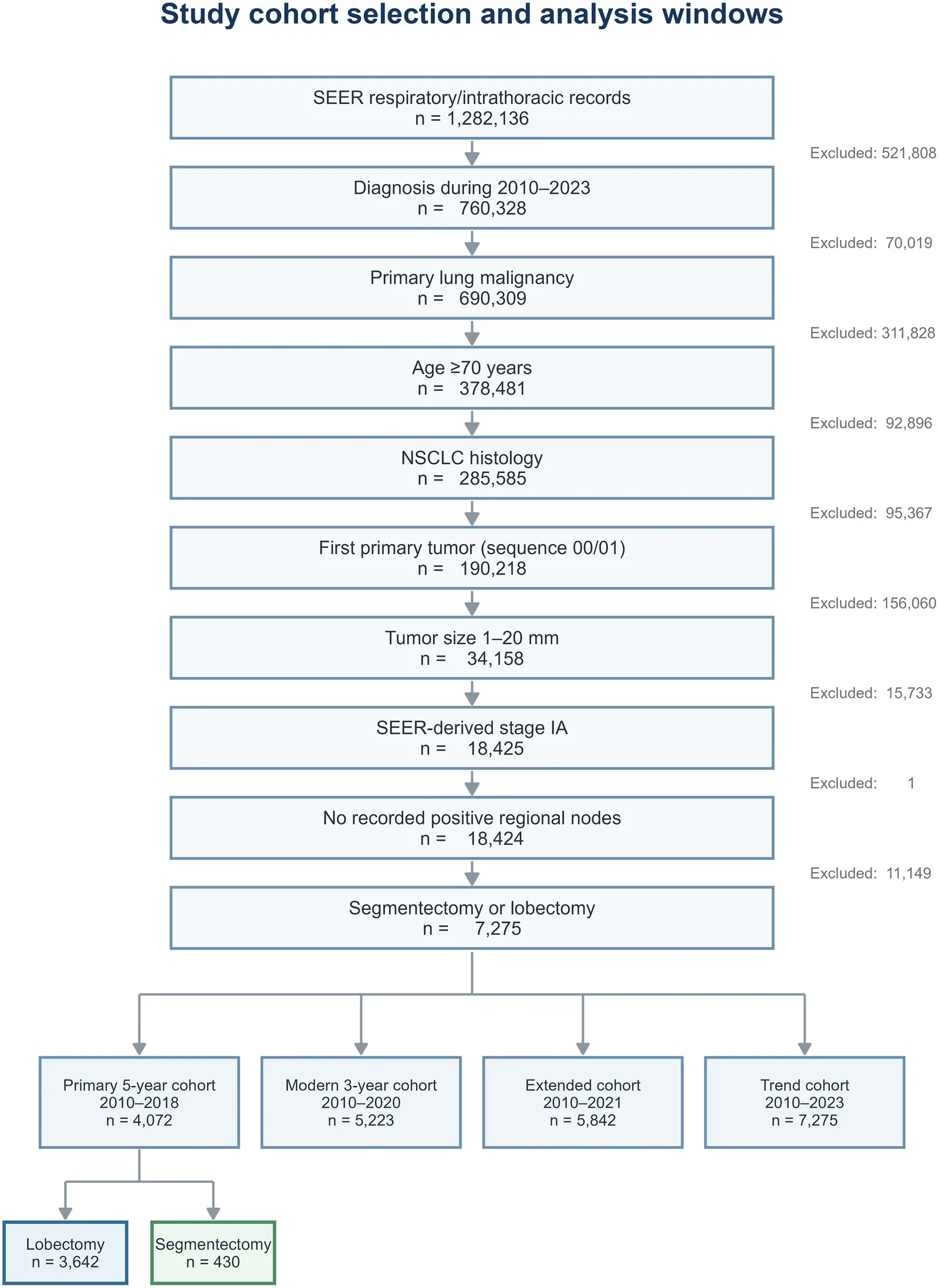
Study cohort selection and analysis windows. The 2010–2023 trend cohort included 7,275 eligible resections; the primary 5-year cohort included diagnoses through 2018, the secondary 3-year cohort through 2020, and the extended time-to-event cohort through 2021.

We used separate diagnosis windows for different follow-up goals. The primary 5-year cohort included patients diagnosed from 2010 through 2018 (*n* = 4,072). The secondary 3-year cohort included diagnoses through 2020 (*n* = 5,223). An extended cohort through 2021 (*n* = 5,842) was used for secondary time-to-event analyses. Patients diagnosed through 2023 were included only in the analysis of surgical use over time, because recent diagnoses did not have sufficient follow-up for long-term mortality analyses.

### 2.3 Stage harmonization and tumor definition

Stage coding changed during the study period. We therefore used the SEER-derived stage group available for each diagnosis era rather than rebuilding stage from individual T, N, and M fields. Stage IA was defined using AJCC 7th-edition-derived stage for 2010–2015, SEER Combined Stage Group for 2016–2017, and EOD 2018/AJCC-derived stage from 2018 onward (22–24). Individual N and M variables were used only to check for major inconsistencies. Tumor size was taken from the cross-era Tumor Size Over Time Recode.

Histology was grouped as adenocarcinoma, squamous-cell carcinoma, or other NSCLC. Primary site was retained at the lung lobe/site level.

### 2.4 Surgical exposure

The exposure was anatomic segmentectomy vs. lobectomy. For cases coded through 2022, surgery code 22 represented segmentectomy/lingulectomy and codes 30 or 33 represented lobectomy/bilobectomy. For 2023, A220 represented segmentectomy and A300/A330 represented lobectomy/bilobectomy. Wedge resection (21 or A210) was excluded. This allowed a direct comparison of anatomic segmental resection with lobar resection.

The number of regional nodes examined was not included in the baseline propensity-score model because nodal evaluation occurs during the surgical episode and may be affected by the operation itself. We therefore reported nodal evaluation separately as a procedural characteristic. Tumor grade was also excluded from the primary propensity model because it is based on pathologic assessment and had missing data.

### 2.5 Outcomes and follow-up

The two main outcomes were lung cancer-specific death and death from causes other than lung cancer. All-cause death was secondary. Cause of death was based on SEER cause-specific and other-cause death classifications (25). Deaths with unresolved cause were counted in all-cause mortality but not assigned to either competing cause. Only 38 deaths (0.93%) had unresolved cause in the primary cohort.

Follow-up was measured from diagnosis in months. We estimated cumulative incidence at 12, 36, and 60 months. The 60-month result was the main long-term estimate, and 36 months was the main contemporary validation point. Absolute risk difference (RD) was calculated as segmentectomy minus lobectomy. A positive RD for lung cancer death therefore indicates higher mortality after segmentectomy.

### 2.6 Covariates and confounding control

The propensity-score model included age, sex, race/ethnicity, marital status, diagnosis year, laterality, tumor size, histologic group, primary lung site, county-level median household income, and rural-urban category. These variables were selected because they were available before treatment and could affect either surgical choice or prognosis. Age and tumor size were modeled with natural cubic splines using three degrees of freedom. Diagnosis year was modeled categorically. Unknown categories were retained when needed.

We estimated the probability of receiving segmentectomy with logistic regression and then applied overlap weights (26). Overlap weighting gives the greatest weight to patients whose measured characteristics make either operation plausible and downweights patients who were very likely to receive only one operation. The resulting estimate is the average treatment effect in the overlap population (ATO). This approach also reduces the influence of extreme propensity scores (26, 27).

We assessed balance with standardized mean differences (SMDs) and considered an absolute SMD <0.10 acceptable. We also examined propensity-score distributions, weight distributions, and effective sample sizes. Because the analysis targets the overlap population, the results are most applicable to older patients who could reasonably have received either segmentectomy or lobectomy based on measured characteristics.

### 2.7 Competing-risk and survival analyses

We estimated weighted cumulative incidence functions for lung cancer death and other-cause death, treating the two outcomes as competing events. We calculated absolute RDs at the prespecified time points. Confidence intervals for the main 5-year and contemporary 3-year RDs were obtained from 1,000 patient-level bootstrap samples. In each bootstrap sample, the propensity score and overlap weights were re-estimated before the outcome was recalculated.

We also fit weighted cause-specific Cox models for lung cancer death and other-cause death and a weighted Cox model for all-cause death. Fine-Gray models were used as a complementary analysis of cumulative incidence in the presence of competing events (28–30). Because cause-specific and Fine-Gray models answer different questions, we interpreted them together with the absolute risks rather than using one model as the sole result.

### 2.8 Temporal validation and sensitivity analyses

We tested the proportional-hazards assumption with scaled Schoenfeld residuals and cox.zph. Kaplan–Meier-transformed time was used for the main test, with identity-transformed time as a sensitivity check. We also fit a time-varying Cox model with a segmentectomy-by-log(time) term and estimated HRs at 12, 24, 36, 48, and 60 months.

We used piecewise cause-specific Cox models with a prespecified 36-month cut point and repeated the analysis with a 24-month cut point. We also performed landmark analyses among patients who were alive and event-free at 36 months and, in a sensitivity analysis, at 24 months. The cut points were chosen before examining the interval-specific estimates. Landmark results were treated as conditional sensitivity analyses, not as new estimates of the original treatment effect.

We assessed whether age modified the association between surgery and outcome by interacting surgery with a spline for continuous age and jointly testing the interaction terms. Age groups of 70–74, 75–79, and ≥80 years were shown only descriptively because the continuous interaction test was primary. Other sensitivity analyses included patients aged ≥75 years, patients aged ≥80 years, adenocarcinoma or squamous-cell carcinoma only, exclusion of survival <1 month, and a 3-month landmark analysis. To quantify sensitivity to residual unmeasured confounding, we calculated *E*-values for the primary lung cancer cause-specific HR using the recommended hazard-ratio-to-risk-ratio conversion for a non-rare outcome, with the rare-outcome approximation retained as a sensitivity calculation (31). SEER does not contain direct measures of pulmonary function, frailty, smoking exposure, detailed cardiopulmonary comorbidity, or formal physiologic fitness (32).

### 2.9 Procedural context and statistical software

For procedural context, we summarized the number of regional nodes examined in the secondary 2010–2020 cohort and reported the proportions with zero, ≥6, and ≥10 nodes. Because nodal evaluation occurs during the surgical episode and may be affected by the operation, surgeon, and intraoperative findings, node count was not included in the baseline propensity-score model. In additional procedural-adequacy sensitivity analyses, we restricted the primary 5-year cohort to patients with ≥1, ≥6, or ≥10 regional nodes examined. Within each restricted cohort, the original baseline propensity-score model was re-estimated, new ATO overlap weights were derived, covariate balance was reassessed, and 5-year cumulative incidence, absolute RDs, and cause-specific HRs were recalculated. Percentile 95% CIs for restricted-cohort RDs were obtained from 1,000 bootstrap replicates with propensity scores and weights re-estimated in each replicate. These analyses were interpreted as post-exposure procedural-restriction sensitivity analyses rather than adjustment for a baseline confounder.

To provide clinical context for competing mortality, we also summarized the SEER COD to site recode among patients classified as dying from causes other than lung cancer. We retained the original SEER cause-of-death labels for audit and grouped major non-cancer causes into clinically interpretable categories. Within each surgical group, both unweighted percentages and overlap-weighted percentages conditional on other-cause death were calculated. These descriptive cause distributions were not treated as causal comparisons.

Analyses were performed in R version 4.4.2. Overlap weights were estimated with WeightIt version 1.7.0, balance was assessed with cobalt version 4.6.3, and survival analyses used survival version 3.8-6. All tests were two-sided with *P* < 0.05 considered statistically significant. Temporal and subgroup analyses were treated as supportive analyses rather than separate confirmatory tests.

## 3 Results

### 3.1 Study population and covariate balance

The sequential eligibility criteria reduced 1,282,136 source records to 7,275 eligible patients aged ≥70 years with a first primary, 1–20 mm, stage IA NSCLC treated with segmentectomy or lobectomy during 2010–2023 (Figure 1). The primary 2010–2018 cohort included 4,072 patients: 3,642 (89.4%) underwent lobectomy and 430 (10.6%) underwent segmentectomy. The contemporary 2010–2020 cohort included 5,223 patients, and the extended 2010–2021 cohort included 5,842. Median follow-up in the primary cohort was 76 months overall, 77 months after lobectomy, and 74 months after segmentectomy.

Before weighting, patients who underwent segmentectomy were slightly older than those who underwent lobectomy (75.99 vs. 75.11 years) and had smaller tumors (13.81 vs. 14.95 mm). Differences were also present in laterality, lobe, and residential characteristics (Table 1). The largest unweighted absolute SMDs were 0.285 for tumor size, 0.194 for age, 0.132 for residence in a metropolitan county with ≥1 million residents, 0.116 for lower-lobe location, and approximately 0.103 for laterality. After overlap weighting, all 59 modeled covariate terms had absolute SMDs <0.10 (Figure 2). The effective sample size was 2,642.7 for lobectomy and 425.8 for segmentectomy.

**Table 1:** Baseline characteristics before and after propensity-score overlap weighting in the primary 2010–2018 cohort.

Characteristic	Lobectomy unweighted	Segmentectomy unweighted	SMD before	Lobectomy weighted	Segmentectomy weighted	SMD after
Age, years	75.11 ± 4.10	75.99 ± 4.61	0.194	75.85 ± 4.53	75.85 ± 4.54	0.000
Male sex	1,522 (41.8%)	164 (38.1%)	0.037	38.5%	38.5%	0.000
Non-Hispanic White	2,899 (79.6%)	355 (82.6%)	0.030	82.2%	82.2%	0.000
Non-Hispanic Black	208 (5.7%)	21 (4.9%)	0.008	5.0%	5.0%	0.000
Non-Hispanic Asian/Pacific Islander	284 (7.8%)	25 (5.8%)	0.020	6.2%	6.2%	0.000
Hispanic	238 (6.5%)	25 (5.8%)	0.007	6.1%	6.1%	0.000
Married	2027 (55.7%)	221 (51.4%)	0.043	52.0%	52.0%	0.000
Widowed	767 (21.1%)	106 (24.7%)	0.036	24.0%	24.0%	0.000
Right-sided primary	2,237 (61.4%)	220 (51.2%)	0.103	52.2%	52.2%	0.000
Tumor size, mm	14.95 ± 3.90	13.81 ± 4.03	0.285	13.98 ± 4.00	13.98 ± 4.01	0.000
Adenocarcinoma	2,648 (72.7%)	308 (71.6%)	0.011	71.9%	71.9%	0.000
Squamous-cell carcinoma	847 (23.3%)	107 (24.9%)	0.016	24.5%	24.5%	0.000
Upper-lobe primary	2,179 (59.8%)	231 (53.7%)	0.061	54.8%	54.8%	0.000
Lower-lobe primary	1,171 (32.2%)	188 (43.7%)	0.116	42.4%	42.4%	0.000
Metropolitan county ≥1 million	2,211 (60.7%)	318 (74.0%)	0.132	72.4%	72.4%	0.000

Values are mean ± SD or *n* (%) before weighting and weighted means/proportions after weighting. SMD, standardized mean difference. Complete baseline model-term balance is provided in Supplementary Table S1; the propensity-score distance is shown separately as a diagnostic rather than a baseline covariate.

**Figure 2:**
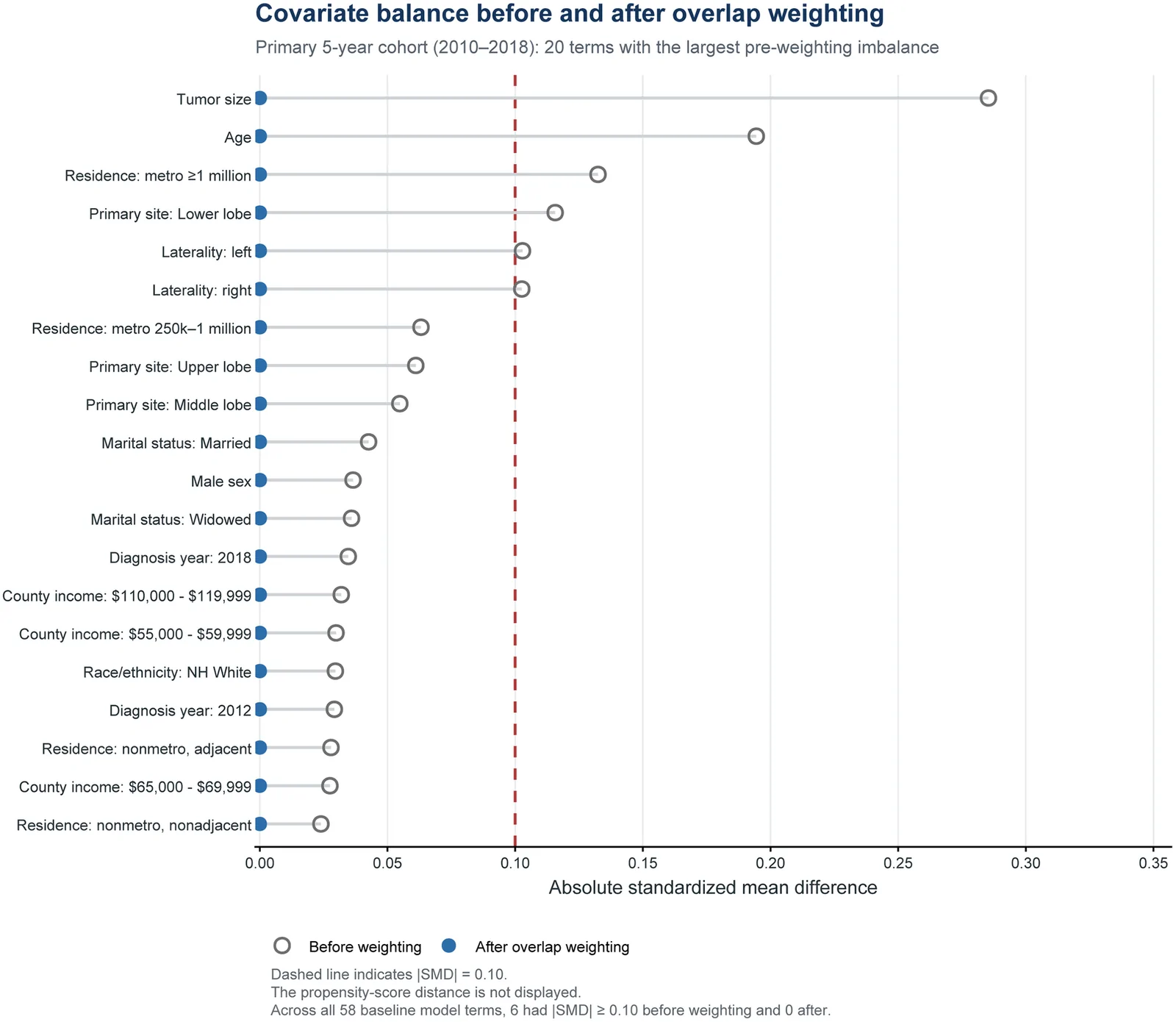
Covariate balance before and after propensity-score overlap weighting in the primary 5-year cohort. Absolute standardized mean differences are shown before and after weighting; all baseline model terms achieved |SMD| <0.10 after weighting.

### 3.2 Surgical adoption and procedural context

Use of segmentectomy increased from 9.1% of eligible resections in 2010 to 24.5% in 2023. Nodal evaluation was less extensive after segmentectomy. In the 2010–2020 cohort, the median number of examined nodes was 9 (IQR, 5–15) after lobectomy and 5 (IQR, 2–11) after segmentectomy. No nodes were examined in 3.2% and 14.9% of patients, respectively. At least 6 nodes were examined in 71.8% after lobectomy and 48.6% after segmentectomy; at least 10 nodes were examined in 48.2% and 30.4%. These results were treated as procedural characteristics and were not included in the baseline propensity model.

### 3.3 Five-year cancer-specific and competing mortality

In the primary cohort, lung cancer-specific mortality was similar early after surgery but differed more at longer follow-up (Figure 3). At 12 months, the weighted cumulative incidence was 2.61% after lobectomy and 1.65% after segmentectomy (RD, −0.95 per 100 patients). At 36 months, the risks were 6.79% and 7.89% (RD, +1.10 per 100). At 60 months, lung cancer-specific mortality was 11.15% after lobectomy and 15.23% after segmentectomy. The 5-year RD was +4.08 lung cancer deaths per 100 patients. All 1,000 bootstrap analyses were successful, and the 95% CI was +0.63 to +7.31 per 100 (Table 2).

**Figure 3:**
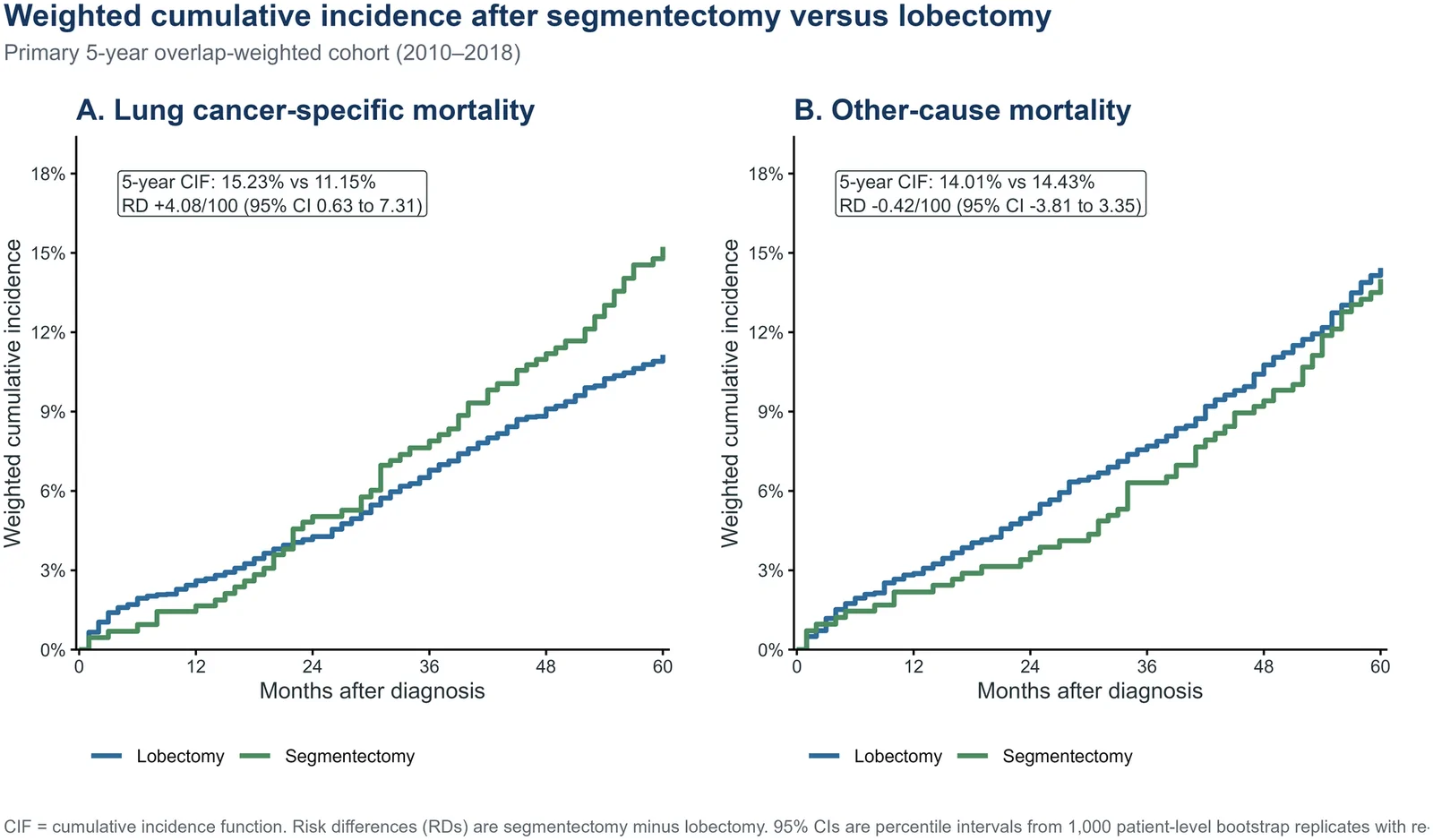
Overlap-weighted cumulative incidence of lung cancer-specific and other-cause mortality after segmentectomy versus lobectomy in the primary 5-year cohort. **(A)** Lung cancer-specific death. **(B)** Other-cause death. At 5 years, the absolute risk difference in lung cancer-specific death was +4.08 deaths per 100 patients (segmentectomy minus lobectomy; 95% percentile bootstrap CI, +0.63 to +7.31). The corresponding other-cause mortality risk difference was −0.42 per 100 patients (95% CI, −3.81 to +3.35).

**Table 2:** Weighted absolute and relative mortality outcomes after segmentectomy vs. lobectomy.

Outcome	Lobectomy	Segmentectomy	Absolute RD per 100 (95% CI)	Relative effect (95% CI)	*P* value
Primary lung cancer death, 1 year	2.61%	1.65%	−0.95		
Primary lung cancer death, 3 years	6.79%	7.89%	1.10		
Primary lung cancer death, 5 years	11.15%	15.23%	4.08 (0.63 to 7.31)		
Primary other-cause death, 5 years	14.43%	14.01%	−0.42 (−3.81 to 3.35)		
Primary lung cancer cause-specific HR	Reference			1.31 (1.05–1.63)	0.015
Primary lung cancer Fine-Gray sHR	Reference			1.30 (1.05–1.62)	0.017
Primary other-cause cause-specific HR	Reference			1.14 (0.95–1.36)	0.161
Primary other-cause Fine-Gray sHR	Reference			1.09 (0.91–1.30)	0.365
Primary all-cause HR	Reference			1.19 (1.04–1.37)	0.011
Modern lung cancer death, 3 years	6.15%	6.77%	0.61 (−1.44 to 2.89)		
Modern other-cause death, 3 years	7.12%	6.19%	−0.92 (−2.91 to 1.18)		

RD is segmentectomy minus lobectomy. Bootstrap confidence intervals were generated for the prespecified 5-year primary and 3-year contemporary RDs with re-estimation of the propensity score and overlap weights in each of 1,000 replicates.

The relative models showed the same pattern. Segmentectomy was associated with a higher cause-specific hazard of lung cancer death (HR, 1.31; 95% CI, 1.05–1.63; *P* = 0.015) and a higher Fine-Gray subdistribution hazard (sHR, 1.30; 95% CI, 1.05–1.62; *P* = 0.017). The all-cause HR was 1.19 (95% CI, 1.04–1.37; *P* = 0.011).

Other-cause mortality did not show the same long-term difference. At 60 months, cumulative other-cause mortality was 14.43% after lobectomy and 14.01% after segmentectomy (RD, −0.42 per 100; 95% bootstrap CI, −3.81 to +3.35). The cause-specific HR was 1.14 (95% CI, 0.95–1.36; *P* = 0.161), and the Fine-Gray sHR was 1.09 (95% CI, 0.91–1.30; *P* = 0.365).

### 3.4 Contemporary validation and temporal pattern

In the contemporary 2010–2020 cohort, the 3-year absolute difference in lung cancer mortality was small. Weighted lung cancer mortality at 36 months was 6.15% after lobectomy and 6.77% after segmentectomy (RD, +0.61 per 100; 95% bootstrap CI, −1.44 to +2.89). The 3-year other-cause RD was −0.92 per 100 (95% CI, −2.91 to +1.18). Neither absolute difference excluded zero.

The relative estimates remained in the same direction. In the contemporary cohort, the cause-specific HR for lung cancer death was 1.26 (95% CI, 1.03–1.56; *P* = 0.027), and the Fine-Gray sHR was 1.25 (95% CI, 1.02–1.54; *P* = 0.034). Other-cause mortality remained nonsignificant. In the extended 2010–2021 cohort, the lung cancer cause-specific HR was 1.29 (95% CI, 1.05–1.59; *P* = 0.015).

There was no evidence that the proportional-hazards assumption was violated. In the primary cohort, cox.zph *P* values for surgery were 0.468 for lung cancer death, 0.454 for other-cause death, and 0.307 for all-cause death. Results were similar with identity-transformed time.

The time-varying analysis showed numerically larger lung cancer HR estimates at later follow-up (Figure 4; Table 3). The estimated HR was 1.12 (95% CI, 0.78–1.61) at 12 months, 1.38 (1.05–1.81) at 24 months, 1.56 (1.17–2.08) at 36 months, 1.70 (1.22–2.36) at 48 months, and 1.82 (1.25–2.64) at 60 months. However, the continuous segmentectomy-by-log(time) interaction did not reach conventional statistical significance (*P* = 0.058), and proportional hazards were not rejected. The corresponding interaction for other-cause mortality was *P* = 0.501. We therefore regarded this temporal pattern as exploratory and hypothesis-generating rather than evidence of a confirmed change in treatment effect over time.

**Figure 4:**
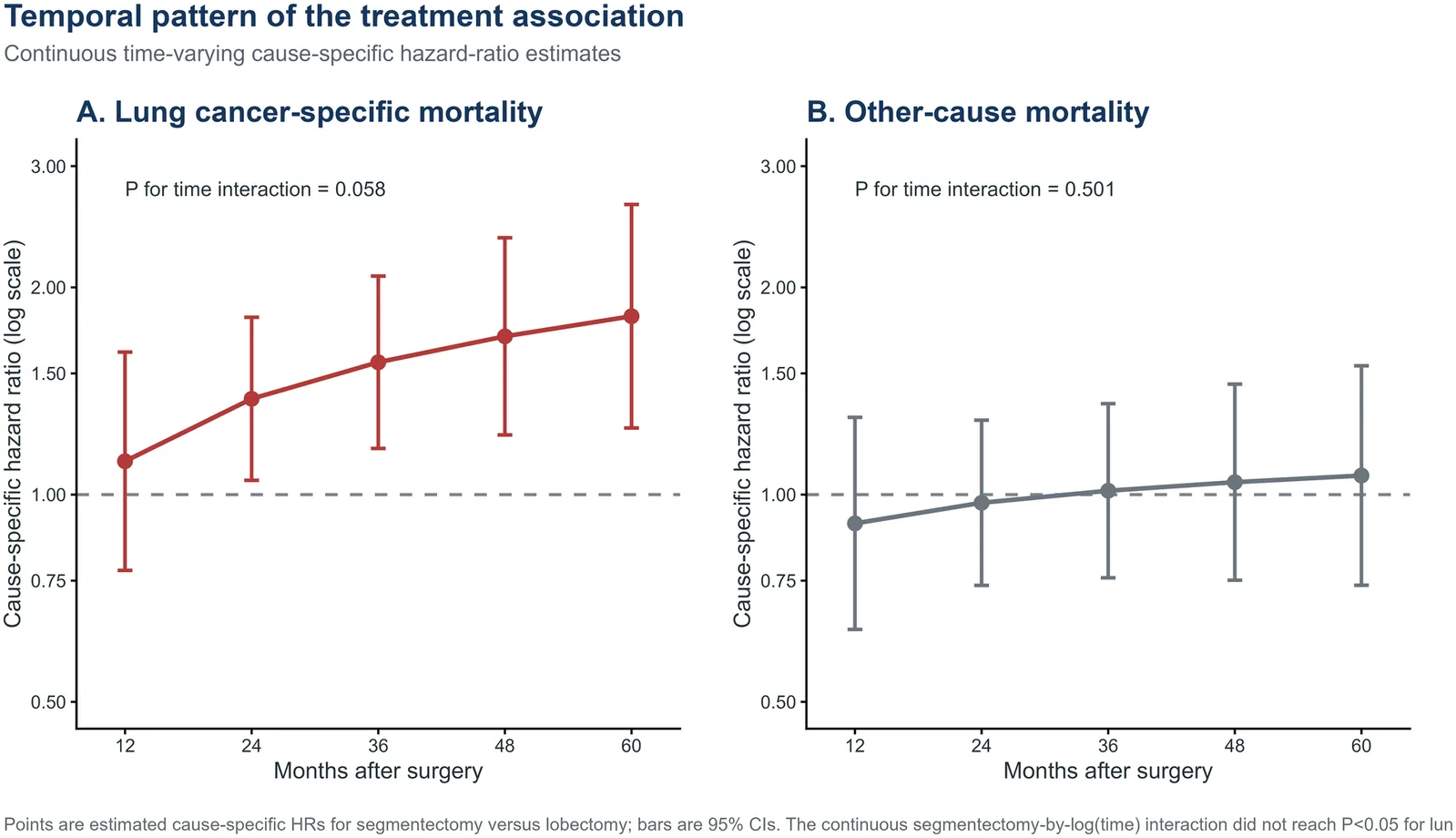
Time-varying cause-specific hazard ratios for segmentectomy versus lobectomy. **(A)** Lung cancer-specific mortality. **(B)** Other-cause mortality. Points indicate hazard ratio estimates at 12, 24, 36, 48, and 60 months, and bars indicate 95% confidence intervals. The continuous treatment-by-time interaction was *P* = 0.058 for lung cancer death and *P* = 0.501 for other-cause death. The temporal pattern is exploratory and does not establish a time-varying treatment effect.

**Table 3:** Temporal and heterogeneity validation of lung cancer-specific mortality.

Analysis	Time/interval	Estimate (95% CI)	*P* value	Role
cox.zph	Primary 5-year cohort	*Χ*^2^ = 0.53	0.468	PH diagnostic
Time-varying Cox	12 months	HR 1.12 (0.78–1.61)		Supportive profile
Time-varying Cox	24 months	HR 1.38 (1.05–1.81)		Supportive profile
Time-varying Cox	36 months	HR 1.56 (1.17–2.08)		Supportive profile
Time-varying Cox	48 months	HR 1.70 (1.22–2.36)		Supportive profile
Time-varying Cox	60 months	HR 1.82 (1.25–2.64)		Supportive profile
Continuous time interaction	Segmentectomy × log(time)	*β* = 0.301	0.058	Supportive; not definitive
Piecewise Cox (36-month cut)	0–36	HR 1.15 (0.79–1.66)	0.464	Sensitivity
Piecewise Cox (36-month cut)	>36–60	HR 1.71 (1.15–2.54)	0.008	Sensitivity
Piecewise Cox (24-month cut)	0–24	HR 1.16 (0.73–1.85)	0.524	Sensitivity
Piecewise Cox (24-month cut)	>24–60	HR 1.49 (1.07–2.08)	0.018	Sensitivity
36-month landmark	36–60 months	HR 1.71 (1.15–2.54); RD 3.44/100	0.008	Conditional sensitivity
24-month landmark	24–60 months	HR 1.49 (1.07–2.08); RD 3.56/100	0.018	Conditional sensitivity
Age interaction	Continuous spline, 3 df	Wald *χ*^2^ = 5.29	0.152	No evidence of modification

The continuous time interaction did not meet *P* < 0.05 and the Schoenfeld test did not reject proportional hazards; piecewise and landmark analyses are therefore interpreted as supportive rather than confirmatory evidence of temporal strengthening.

Piecewise analyses provided supportive but nonconfirmatory context. The lung cancer HR was 1.15 (95% CI, 0.79–1.66; *P* = 0.464) during the first 36 months and 1.71 (1.15–2.54; *P* = 0.008) from >36 to 60 months. With a 24-month cut point, the HR was 1.16 (0.73–1.85; *P* = 0.524) during 0–24 months and 1.49 (1.07–2.08; *P* = 0.018) during >24–60 months. Because the formal time interaction was *P* = 0.058 and the proportional-hazards assumption was not rejected, these interval-specific findings were treated as exploratory sensitivity analyses rather than evidence of a discrete delayed hazard transition.

### 3.5 Landmark validation and age heterogeneity

Among 3,454 patients who were alive and event-free at 36 months, subsequent lung cancer mortality through 60 months was higher after segmentectomy (HR, 1.71; 95% CI, 1.15–2.54; *P* = 0.008). The conditional RD was +3.44 lung cancer deaths per 100 patients. Other-cause mortality did not differ significantly. The 24-month landmark analysis gave a similar result for lung cancer death (HR, 1.49; 95% CI, 1.07–2.08; *P* = 0.018; conditional RD, +3.56 per 100). The piecewise and landmark sensitivity estimates are shown in Figure 5 (panels A and B, respectively).

**Figure 5:**
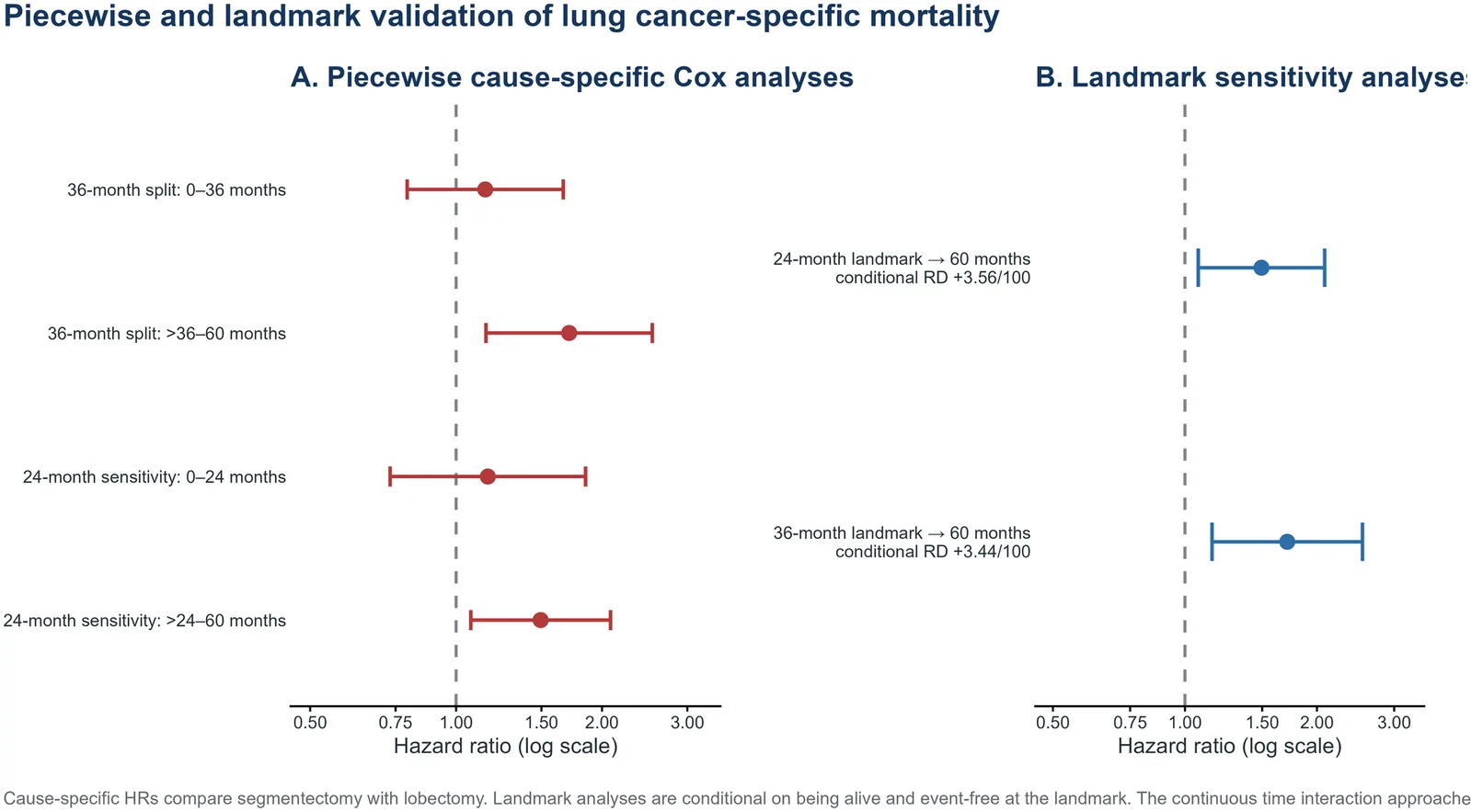
Piecewise and landmark analyses of lung cancer-specific mortality after segmentectomy versus lobectomy. **(A)** Piecewise cause-specific Cox estimates using 36-month and 24-month cut points. **(B)** Landmark analyses at 24 and 36 months among patients who remained alive and event-free at each landmark. Error bars indicate 95% confidence intervals. Landmark estimates are conditional sensitivity analyses and should not be interpreted as baseline causal treatment effects.

Age did not significantly modify the association between surgery and mortality. The global interaction *P* value was 0.152 for lung cancer death and 0.859 for other-cause death. We therefore treated age-stratified estimates as descriptive rather than evidence of an age-dependent treatment effect.

### 3.6 Residual-confounding, nodal-adequacy, and cause-of-death sensitivity analyses

The *E*-value analysis indicated limited-to-moderate robustness to unmeasured confounding. For the primary lung cancer cause-specific HR of 1.31 (95% CI, 1.05–1.63), the *E*-value was 1.71 for the point estimate and 1.23 for the confidence-limit estimate using the non-rare-outcome HR-to-RR conversion. The corresponding rare-outcome sensitivity values were 1.95 and 1.29 (Supplementary Table S7). These values indicate that residual confounding of modest magnitude could attenuate the observed association.

Procedural-adequacy-restricted analyses showed progressive attenuation with more stringent nodal evaluation (Supplementary Table S8). Among patients with ≥1 examined node (*n* = 3,618; 330 segmentectomies), the 5-year lung cancer RD remained +4.00 per 100 patients (95% percentile bootstrap CI, +0.17 to +8.04), and the cause-specific HR was 1.27 (95% CI, 0.99–1.64; *P* = 0.060). With ≥6 examined nodes (*n* = 2,526; 168 segmentectomies), the RD was +4.18 per 100 (95% CI, −1.04 to +9.58) and the HR was 1.12 (95% CI, 0.76–1.67; *P* = 0.563). With ≥10 examined nodes (*n* = 1,626; 98 segmentectomies), the RD attenuated to +1.88 per 100 (95% CI, −4.58 to +8.59) and the HR was 1.00 (95% CI, 0.61–1.65; *P* = 0.996). The increasingly strict restrictions substantially reduced the segmentectomy sample and precision.

Other-cause mortality estimates shifted below the null under the more stringent nodal restrictions, but these analyses condition on a procedure-related post-exposure characteristic and involved small segmentectomy samples; they were therefore considered exploratory. In the primary cohort, the leading overlap-weighted categories among other-cause deaths were cardiovascular disease (26.8% after lobectomy and 30.2% after segmentectomy) and COPD/chronic respiratory disease (14.0% and 22.5%, respectively), followed by unspecified/other causes and cerebrovascular disease (Supplementary Table S9). These descriptive percentages characterize the composition of competing deaths and do not estimate causal effects of surgical extent on specific non-cancer causes.

## 4 Discussion

### 4.1 Main findings

This study found two main results. First, in the primary overlap-weighted analysis, segmentectomy was associated with higher lung cancer-specific mortality at 5 years in older patients with small stage IA NSCLC, corresponding to 4.08 additional lung cancer deaths per 100 patients. Second, segmentectomy did not show a clear overall reduction in death from other causes. However, the new sensitivity analyses materially qualify the first finding: the *E*-values were modest, and the cancer-specific association attenuated as analyses were restricted to patients with more extensive nodal evaluation. These results suggest that patient selection and procedural oncologic quality may contribute to the real-world association and argue against interpreting the primary estimate as an intrinsic causal disadvantage of segmentectomy.

### 4.2 Comparison with randomized and large clinical studies

The randomized trials provide the most important context for these results. JCOG0802/WJOG4607L showed that segmentectomy can achieve excellent survival in selected peripheral NSCLC ≤2 cm and even improved overall survival compared with lobectomy, but local relapse was more frequent after segmentectomy (7). CALGB/Alliance 140503 showed that sublobar resection was noninferior to lobectomy for disease-free survival when tumors were small, peripheral, and carefully staged as node-negative (8). A post hoc analysis of CALGB/Alliance 140503 also evaluated perioperative morbidity and mortality after sublobar versus lobar resection (33). JCOG1211 showed very good outcomes after segmentectomy for ground-glass-dominant tumors (9). A prospective randomized multicenter phase III study also compared anatomical segmentectomy with standard lobectomy for non-small cell lung cancer measuring up to 2 cm (34). These trials support segmentectomy when the tumor and the operation meet strict criteria.

Our study does not overturn those trials. It addresses a different population and a different outcome question. Trial patients are highly selected, surgical quality is controlled, and nodal staging is protocol driven. SEER reflects routine practice across many hospitals and surgeons and includes older patients with a wider range of health status. In addition, our main question was not simply whether overall survival differed. We separated lung cancer death from death due to other causes and reported absolute differences at 3 and 5 years.

### 4.3 Why our findings differ from some trial signals

The clearest difference from recent JCOG0802 analyses concerns other-cause mortality. In the 2026 *post hoc* analysis of patients with pathologically invasive features, segmentectomy was associated with fewer deaths from other causes, while lung cancer-specific death did not differ significantly (19). That result supports the idea that preserving lung tissue may improve long-term survival through better physiologic reserve. We did not see a clear reduction in other-cause mortality in the SEER cohort.

There are several possible reasons. Segmentectomy preserves more pulmonary parenchyma and generally more postoperative pulmonary function than lobectomy (11, 35, 36), but the clinical value of that preservation depends strongly on baseline physiologic reserve. In routine practice, especially during the earlier years of this cohort, surgeons may have selected segmentectomy for patients with poorer pulmonary function, frailty, severe cardiopulmonary disease, or limited tolerance for lobectomy. SEER does not record FEV1, DLCO, ECOG performance status, frailty, smoking exposure, or detailed comorbidity. Overlap weighting balanced the measured baseline variables but could not balance these factors. The *E*-value analysis reinforces this limitation: the *E*-value was 1.71 for the point estimate and only 1.23 for the confidence limit closest to the null, indicating that unmeasured confounding of modest magnitude could materially attenuate the observed association. The primary result should therefore be interpreted as a real-world association rather than evidence that segmentectomy itself increases cancer mortality.

### 4.4 Possible reasons for the higher long-term lung cancer mortality

The lung cancer result also needs to be interpreted in the context of previous clinical evidence. JCOG0802 reported more local relapse after segmentectomy (7), and later analyses showed that recurrence risk is concentrated in some tumor subgroups (18, 19). In our study, the 3-year absolute difference was small and the 5-year difference was larger, but this should not be described as a confirmed delayed divergence. The formal surgery-by-time interaction was *P* = 0.058 and proportional hazards were not rejected. The larger separation observed later in follow-up is therefore best considered a hypothesis-generating temporal pattern rather than evidence of a proven transition in treatment effect.

The quality of the oncologic procedure may also contribute. Segmentectomy requires an appropriate tumor, adequate parenchymal margins, and systematic hilar and mediastinal nodal assessment (37). National Cancer Database research has shown that compliance with segmentectomy quality measures is variable and associated with survival (38), and randomized trials such as CALGB 140503 mandated rigorous nodal evaluation before limited resection (8). In our secondary cohort, nodal assessment was clearly less extensive after segmentectomy (39). The new restriction analyses are informative in this context. The 5-year lung cancer RD remained approximately +4 per 100 among patients with ≥1 or ≥6 examined nodes, but the ≥6-node confidence interval included zero and the cause-specific HR attenuated to 1.12. With ≥10 examined nodes, the RD fell to +1.88 per 100 and the HR to 1.00, although only 98 segmentectomy patients remained. These findings do not prove that nodal evaluation mediates the primary association, because node count is a post-exposure procedural characteristic and increasingly strict restrictions select a smaller subgroup. They do, however, suggest that differences in real-world procedural quality and understaging may contribute to the primary estimate.

Margins may also matter. Recent studies have linked larger margins after thoracoscopic segmentectomy with better locoregional control (40), although other carefully selected cohorts have reported low recurrence even with closer margins (41, 42). SEER does not provide margin distance or recurrence site, so we cannot test whether inadequate margins explain the mortality difference.

Tumor biology is another major source of unresolved heterogeneity. STAS is associated with recurrence in stage I adenocarcinoma (43), and a propensity-matched analysis found better outcomes with lobectomy than sublobar resection in STAS-positive T1 adenocarcinoma (44). In contrast, ground-glass-dominant tumors have very favorable outcomes after segmentectomy (9). SEER does not record ground-glass proportion, consolidation-to-tumor ratio, STAS, PET avidity, visceral pleural or lymphovascular invasion in the detail needed for modern surgical selection, or recurrence patterns. The present cohort therefore combines biologically favorable and higher-risk small tumors that would be treated differently in contemporary protocol-driven practice. Our findings should not be extrapolated directly to carefully selected ground-glass-dominant or otherwise favorable tumors undergoing intentional, oncologically complete segmentectomy.

These limitations help explain why the result of a population-based study may differ from a trial. Randomized studies show what can be achieved with careful selection and controlled surgery. Our study shows what was observed across routine practice in older patients. The two types of evidence answer different but complementary questions.

### 4.5 How this study adds to previous observational evidence

Previous SEER studies in older patients have reported mixed results (13–15). A meta-analysis also concluded that segmentectomy can provide survival similar to lobectomy in selected elderly patients, but most included studies were retrospective (16). A separate systematic review and meta-analysis compared perioperative and survival outcomes after segmentectomy versus lobectomy in stage IA NSCLC (45). More recently, a 2026 SEER study of stage IA lung adenocarcinoma ≤20 mm found no significant mortality difference after propensity-score matching (17). Earlier single-institution studies evaluated intentional limited resection in selected patients with T1N0M0 NSCLC and segmentectomy with lymph node assessment for tumors measuring 2 cm or smaller (46, 47). Comparative studies have also examined anatomical segmentectomy versus wedge resection in stage IA NSCLC (48) and lobectomy versus limited resection for stage I NSCLC measuring 1 cm or smaller using SEER data (49). Propensity-score-matched studies have compared segmentectomy with lobectomy in clinical stage IA lung adenocarcinoma (50) and clinical stage I NSCLC (51). Our study adds several pieces of information that were not usually examined together in these reports.

First, we focused specifically on adults aged ≥70 years, in whom competing mortality is a major part of treatment decisions. Second, we separated lung cancer death from other-cause death instead of relying mainly on overall survival. Third, we reported absolute risk differences, which are easier to translate into clinical discussions than hazard ratios alone. The primary 5-year result corresponded to approximately four additional lung cancer deaths per 100 patients after segmentectomy in the overlap population. Fourth, we directly examined how that association changed under increasingly stringent nodal-evaluation restrictions. Finally, overlap weighting kept almost all segmentectomy patients while focusing the comparison on patients for whom either operation was plausible based on measured characteristics. Together with the *E*-value analysis, these additions make the limits of causal interpretation more explicit.

These features define the main contribution of the study. We tested a specific clinical hypothesis raised by modern trials: whether greater parenchymal preservation in older patients is accompanied by fewer competing deaths, and whether routine-practice cancer-specific outcomes differ between operations. In the primary cohort, a 5-year cancer-specific association was observed without a clear overall competing-mortality advantage. However, attenuation under more extensive nodal evaluation and modest *E*-values indicate that the primary association is likely influenced, at least in part, by patient selection and procedural context. The study therefore complements rather than contradicts randomized evidence from carefully selected and protocol-treated patients.

### 4.6 Clinical implications, strengths, and limitations

The results do not support using age alone to choose segmentectomy. An older patient with poor pulmonary reserve, frailty, limited life expectancy, or a favorable ground-glass-dominant tumor may still be an excellent candidate for segmentectomy. A physiologically fit older patient with a solid or otherwise higher-risk tumor may reasonably undergo lobectomy if it can be performed safely. For patients who could receive either operation, the decision should include pulmonary function, frailty, imaging features, expected margin, nodal strategy, life expectancy, and patient preference. This is also consistent with current guideline-based surgical decision making (5, 32).

The study has several strengths. It used a large population-based registry, compared anatomic segmentectomy directly with lobectomy, and used stage definitions appropriate to each SEER era. Overlap weighting achieved excellent measured balance and retained almost the entire segmentectomy group. We reported absolute risks, cause-specific Cox models, Fine-Gray models, and bootstrap confidence intervals. The secondary cohort, extended cohort, landmark analyses, age-interaction analysis, *E*-values, and nodal-adequacy restrictions were used to examine whether the primary association was sensitive to follow-up window, analytic method, unmeasured-confounding assumptions, or procedural selection.

The main limitation is residual confounding by indication. SEER does not record pulmonary function, frailty, detailed cardiopulmonary comorbidity, smoking exposure, performance status, or postoperative treatment tolerance, all of which can influence both the decision to perform segmentectomy and subsequent outcomes. The modest *E*-values show that confounding of plausible magnitude could materially attenuate the primary association. SEER also lacks consolidation-to-tumor ratio, ground-glass morphology, STAS, detailed invasive pathologic features, margin distance, recurrence, and surgeon or hospital characteristics. The nodal-restriction analyses further suggest that procedural oncologic quality may contribute to the observed association; however, because nodes examined is post-exposure and the stricter restrictions markedly reduced the number of segmentectomy patients, these analyses cannot isolate a causal effect of nodal assessment. Cause of death can also be misclassified in registry data, although SEER uses standardized algorithms (25). Stage definitions changed over time, and some stage migration may remain despite era-specific stage harmonization (22–24). Finally, overlap weighting estimates the association in patients with measured characteristics compatible with either operation and should not be generalized to all potentially eligible patients. The temporal analyses were exploratory: proportional hazards were not rejected and the continuous time interaction was *P* = 0.058. Future studies should combine registry-scale follow-up with pulmonary function, frailty, smoking, comorbidity, radiologic phenotype, margin, nodal-station, recurrence, and treatment data.

## 5 Conclusion

Among adults aged ≥70 years with a first primary, 1–20 mm, stage IA NSCLC treated in routine US practice, segmentectomy was associated with higher 5-year lung cancer-specific mortality in the primary overlap-weighted analysis, while overall other-cause mortality was similar. However, the cancer-specific association attenuated under increasingly extensive nodal-evaluation restrictions, and *E*-values indicated sensitivity to unmeasured confounding of modest magnitude. Because SEER lacks pulmonary function, frailty, detailed comorbidity, radiologic tumor phenotype, margin, STAS, and recurrence information, these findings should not be interpreted as evidence of an intrinsic causal disadvantage of segmentectomy in appropriately selected patients. Surgical extent should be individualized according to physiologic reserve, tumor biology, margin feasibility, nodal strategy, life expectancy, and patient preference rather than chronological age alone.

## Data Availability

The data analyzed in this study were obtained from the Surveillance, Epidemiology, and End Results (SEER) Program of the National Cancer Institute, subject to the program's access requirements and the applicable Research Data Use Agreement. The authors are not permitted to redistribute individual-level SEER data. Further inquiries may be directed to the corresponding author.
