# Supplementary material for: Real-world surgical outcomes of segmentectomy vs. lobectomy in older adults with small stage IA NSCLC: long-term cancer-specific and competing mortality in an overlap-weighted SEER cohort
Source: Front Med (Lausanne). 2026 Oct 27;13:1976284. doi: 10.3389/fmed.2026.1976284 (PMC13748310; doi:10.3389/fmed.2026.1976284)
Supplement: Supplementary file 1 [file Supplementaryfile1.docx]

**Supplementary Materials**

**Long-Term Cancer-Specific and Competing Mortality After Segmentectomy Versus Lobectomy in Older Adults With Small Stage IA NSCLC: An overlap-weighted SEER cohort study**

Supplementary Methods

S1. Data source and cohort construction

This study used deidentified population-based data from the Surveillance, Epidemiology, and End Results (SEER) program through 2023. The formal cohort was restricted to diagnoses from 2010 onward to provide consistent contemporary staging information and to define prespecified follow-up windows. The source export contained 1,282,136 respiratory and intrathoracic malignancy records. Sequential restrictions were diagnosis during 2010–2023, primary lung malignancy, age ≥70 years, non-small cell lung cancer (NSCLC) histology, first primary malignancy (SEER sequence 00 or 01), tumor size 1–20 mm, and stage IA disease. One record with a recorded positive regional lymph node was excluded during the final eligibility audit. Patients were then restricted to anatomic segmentectomy or lobectomy, yielding 7,275 eligible resections for the 2010–2023 utilization cohort.

Three survival cohorts were prespecified according to the amount of calendar follow-up available. The primary 5-year cohort included diagnoses during 2010–2018 (n=4,072; 3,642 lobectomies and 430 segmentectomies) and was used for 60-month absolute-risk estimation. The secondary 3-year cohort included diagnoses during 2010–2020 (n=5,223; 4,619 lobectomies and 604 segmentectomies) and was used for the 36-month validation horizon. The extended time-to-event cohort included diagnoses during 2010–2021 (n=5,842; 5,135 lobectomies and 707 segmentectomies). The full 2010–2023 cohort was used only for temporal utilization analyses.

S2. Stage harmonization and tumor definitions

Stage IA eligibility was based on the SEER-derived stage group appropriate to each diagnosis era rather than reconstructing a single TNM system retrospectively. For 2010–2015, the AJCC 7th-edition-derived stage group was used; for 2016–2017, the SEER Combined Stage Group was used; and from 2018 onward, the EOD 2018/AJCC-derived SEER stage group was used. Accepted stage tokens included IA, IA1, IA2, IA3 and the corresponding numeric forms 1A, 1A1, 1A2, and 1A3. Era-specific N and M variables were used as audit fields rather than redundant eligibility filters. Tumor size was preferentially obtained from the SEER Tumor Size Over Time Recode and restricted to 1–20 mm.

NSCLC histology was grouped as adenocarcinoma, squamous-cell carcinoma, or other NSCLC. Small-cell carcinoma, carcinoid/neuroendocrine tumors, large-cell neuroendocrine carcinoma, mesothelioma, sarcoma, melanoma, and hematologic malignancies were excluded by the formal cohort builder. Primary tumor site and laterality were retained as modeling variables.

S3. Surgical exposure and procedural variables

The exposure was anatomic segmentectomy versus lobectomy. For surgery codes used through 2022, code 22 represented segmentectomy/lingulectomy and codes 30 or 33 represented lobectomy/bilobectomy. For 2023, A220 represented segmentectomy and A300/A330 represented lobectomy/bilobectomy. Wedge resection (21 or A210) was excluded, so the comparison did not combine anatomic segmentectomy with nonanatomic wedge resection.

Regional nodes examined was treated as a procedure-related characteristic rather than a baseline confounder because nodal evaluation may be influenced by the operation, surgeon, and intraoperative findings. It was therefore excluded from the primary propensity-score model and summarized separately as procedural context. Pathologic grade was also excluded from the primary propensity model because it is post-resection pathologic information and had nontrivial missingness.

S4. Outcomes and follow-up

The two competing cause-specific outcomes were lung cancer-specific death and death from causes other than lung cancer. All-cause death was a secondary outcome. Cause classification was derived from SEER cause-specific and other-cause death variables. Deaths with unresolved cause classification were not assigned to either competing event but were retained for all-cause mortality. Follow-up was measured in months from diagnosis; registry-coded 0-month survival was represented as 0.5 month for time-to-event analyses rather than discarded.

The primary absolute-risk estimands were overlap-weighted cumulative incidences at 12, 36, and 60 months in the primary cohort. The 60-month estimate was the principal long-term endpoint. In the secondary cohort, cumulative incidence was estimated at 12, 24, and 36 months, with 36 months as the principal validation horizon. Absolute risk difference (RD) was defined as weighted risk after segmentectomy minus weighted risk after lobectomy; therefore, a positive RD indicates a higher cumulative incidence after segmentectomy.

S5. Propensity-score overlap weighting

The propensity for segmentectomy was estimated by logistic regression. The primary estimand was the average treatment effect in the overlap population (ATO). For patients who underwent segmentectomy, the overlap weight was proportional to 1 minus the propensity score; for patients who underwent lobectomy, the overlap weight was proportional to the propensity score. This estimand emphasizes patients with the greatest empirical treatment equipoise and downweights patients with near-deterministic treatment assignment.

The propensity-score model included age, sex, race/ethnicity, marital status, diagnosis year, laterality, tumor size, histologic group, county-level household income, rural–urban category, and primary tumor site. Age and tumor size were modeled with natural cubic splines using three degrees of freedom, and diagnosis year was modeled categorically. Missing categorical values were retained as explicit “Unknown” levels when present. Regional nodes examined and grade were not included in the primary model. Covariate balance was evaluated using standardized mean differences (SMDs), with |SMD|<0.10 prespecified as acceptable. Propensity-score overlap, weight distributions, and effective sample sizes (ESSs) were used as positivity and precision diagnostics.

S6. Competing-risk and relative-effect analyses

Overlap-weighted cumulative incidence functions were estimated in a multistate framework with lung cancer death and other-cause death treated as mutually competing events. For each surgical group, weighted state probabilities were obtained from survival curves using the overlap weights. Risk differences were then calculated directly from the group-specific cumulative incidences.

Percentile 95% confidence intervals for the 3-year and 5-year RDs were obtained from 1,000 nonparametric patient-level bootstrap samples. The propensity-score model and overlap weights were re-estimated within every bootstrap replicate so that uncertainty from treatment weighting was propagated into the RD interval. All 1,000 replicates were successful for both horizons.

Relative associations were summarized using overlap-weighted cause-specific Cox models for lung cancer death and other-cause death, as well as a weighted Cox model for all-cause mortality. Robust standard errors were used. Complementary Fine–Gray analyses were implemented by expanding the competing-risk data and fitting weighted Cox models to the Fine–Gray risk sets. Cause-specific and subdistribution hazard ratios were interpreted as complementary measures and were not treated as interchangeable with absolute risks. Restricted mean time lost to each cause was also calculated over the prespecified 60-month and 36-month horizons as a secondary descriptive measure.

S7. Proportional-hazards and temporal validation

The proportional-hazards assumption was assessed with scaled Schoenfeld residuals using cox.zph. The Kaplan–Meier transformation of time was the primary diagnostic; identity-transformed time was used as a sensitivity analysis in the primary 5-year cohort. No primary or secondary cox.zph test rejected proportional hazards at the conventional 0.05 level.

A supportive continuous time-varying cause-specific Cox analysis was then fitted using a segmentectomy-by-log(time) term, with log time defined as log(max[t, 0.5]). Hazard ratios were evaluated at 12, 24, 36, 48, and 60 months. Because the continuous time interaction for lung cancer mortality was P=0.058, these estimates were interpreted as a supportive time profile rather than proof of statistically significant nonproportional hazards. No time-varying Fine–Gray model was fitted.

Piecewise cause-specific Cox models used a prespecified 36-month cut point to compare 0–36 months with >36–60 months. A 24-month cut point was examined as a sensitivity analysis. The cut points were not selected by maximizing a test statistic. Conditional landmark analyses were performed among patients alive and event-free at 36 or 24 months, with subsequent follow-up through 60 months. Landmark estimates were treated as conditional sensitivity analyses and not as causal effects defined at the original treatment decision.

S8. Age heterogeneity and additional sensitivity analyses

Potential effect modification by age was assessed by interacting surgical treatment with a continuous spline representation of age and jointly testing the three interaction degrees of freedom with a Wald test. Because the global interaction test was primary, risk differences within age groups 70–74, 75–79, and ≥80 years were treated as descriptive. Additional sensitivity analyses repeated 36-month absolute-risk estimation among patients aged ≥75 years, patients aged ≥80 years, patients with adenocarcinoma or squamous-cell carcinoma only, patients surviving at least 1 month, and patients surviving at least 3 months with follow-up re-zeroed at the 3-month landmark.

S9. Procedural context and surgical utilization

Regional-node evaluation was summarized in the 2010–2020 cohort using the median and interquartile range of examined nodes, the proportion with zero examined nodes, and the proportions with ≥6 and ≥10 examined nodes. Event counts were additionally tabulated across categories of 0, 1–5, ≥6, and unknown nodes examined. These analyses were descriptive and were not used to identify a causal mediator. Annual use of segmentectomy and lobectomy among eligible resections was calculated from 2010 through 2023.

S10. Statistical software and interpretation

Analyses were conducted in R version 4.4.2. Propensity-score overlap weights were estimated with WeightIt, balance diagnostics with cobalt, and survival/competing-risk analyses with the survival package; data management used data.table and dplyr. Two-sided P<0.05 was considered statistically significant for primary inferential analyses. Temporal, landmark, age-stratified, and additional sensitivity analyses were interpreted primarily for robustness and pattern consistency and were not treated as multiplicity-adjusted confirmatory tests. Because the target estimand was ATO, treatment-effect estimates apply most directly to older patients whose measured characteristics were compatible with either segmentectomy or lobectomy rather than to every surgically eligible patient.

Supplementary Table S1. Complete model-term balance before and after propensity-score overlap weighting in the primary 5-year cohort.

| Variable/model term | Type | \|SMD\| before weighting | \|SMD\| after weighting |
| --- | --- | --- | --- |
| Propensity-score distance (diagnostic) | Distance | 0.641 | 0.008 |
| Age | Contin. | 0.194 | 0.000 |
| Male sex | Binary | 0.037 | 0.000 |
| Race/ethnicity: Hispanic (All Races) | Binary | 0.007 | 0.000 |
| Race/ethnicity: Non-Hispanic American Indian/Alaska Native | Binary | 0.000 | 0.000 |
| Race/ethnicity: Non-Hispanic Asian or Pacific Islander | Binary | 0.020 | 0.000 |
| Race/ethnicity: Non-Hispanic Black | Binary | 0.008 | 0.000 |
| Race/ethnicity: Non-Hispanic Unknown Race | Binary | 0.006 | 0.000 |
| Race/ethnicity: Non-Hispanic White | Binary | 0.030 | 0.000 |
| Marital status: Divorced | Binary | 0.012 | 0.000 |
| Marital status: Married (including common law) | Binary | 0.043 | 0.000 |
| Marital status: Separated | Binary | 0.000 | 0.000 |
| Marital status: Single (never married) | Binary | 0.001 | 0.000 |
| Marital status: Unknown | Binary | 0.015 | 0.000 |
| Marital status: Unmarried or Domestic Partner | Binary | 0.002 | 0.000 |
| Marital status: Widowed | Binary | 0.036 | 0.000 |
| Diagnosis year: 2010 | Binary | 0.015 | 0.000 |
| Diagnosis year: 2011 | Binary | 0.008 | 0.000 |
| Diagnosis year: 2012 | Binary | 0.029 | 0.000 |
| Diagnosis year: 2013 | Binary | 0.016 | 0.000 |
| Diagnosis year: 2014 | Binary | 0.001 | 0.000 |
| Diagnosis year: 2015 | Binary | 0.007 | 0.000 |
| Diagnosis year: 2016 | Binary | 0.015 | 0.000 |
| Diagnosis year: 2017 | Binary | 0.005 | 0.000 |
| Diagnosis year: 2018 | Binary | 0.035 | 0.000 |
| Laterality: left | Binary | 0.103 | 0.000 |
| Laterality: paired/unknown | Binary | 0.000 | 0.000 |
| Laterality: right | Binary | 0.103 | 0.000 |
| Tumor size (mm) | Contin. | 0.285 | 0.000 |
| Histology: adenocarcinoma | Binary | 0.011 | 0.000 |
| Histology: squamous-cell carcinoma | Binary | 0.016 | 0.000 |
| Histology: other NSCLC | Binary | 0.005 | 0.000 |
| County household income: $100,000 - $109,999 | Binary | 0.019 | 0.000 |
| County household income: $110,000 - $119,999 | Binary | 0.032 | 0.000 |
| County household income: $120,000+ | Binary | 0.011 | 0.000 |
| County household income: $40,000 - $44,999 | Binary | 0.005 | 0.000 |
| County household income: $45,000 - $49,999 | Binary | 0.008 | 0.000 |
| County household income: $50,000 - $54,999 | Binary | 0.006 | 0.000 |
| County household income: $55,000 - $59,999 | Binary | 0.030 | 0.000 |
| County household income: $60,000 - $64,999 | Binary | 0.000 | 0.000 |
| County household income: $65,000 - $69,999 | Binary | 0.027 | 0.000 |
| County household income: $70,000 - $74,999 | Binary | 0.004 | 0.000 |
| County household income: $75,000 - $79,999 | Binary | 0.011 | 0.000 |
| County household income: $80,000 - $84,999 | Binary | 0.006 | 0.000 |
| County household income: $85,000 - $89,999 | Binary | 0.018 | 0.000 |
| County household income: $90,000 - $94,999 | Binary | 0.012 | 0.000 |
| County household income: $95,000 - $99,999 | Binary | 0.010 | 0.000 |
| County household income: < $40,000 | Binary | 0.004 | 0.000 |
| Rural–urban category: Metropolitan ≥1 million | Binary | 0.132 | 0.000 |
| Rural–urban category: Metropolitan 250,000–1 million | Binary | 0.063 | 0.000 |
| Rural–urban category: Metropolitan <250,000 | Binary | 0.018 | 0.000 |
| Rural–urban category: Nonmetropolitan, adjacent | Binary | 0.028 | 0.000 |
| Rural–urban category: Nonmetropolitan, nonadjacent | Binary | 0.024 | 0.000 |
| Primary site: C340 main bronchus | Binary | 0.001 | 0.000 |
| Primary site: C341 upper lobe | Binary | 0.061 | 0.000 |
| Primary site: C342 middle lobe | Binary | 0.055 | 0.000 |
| Primary site: C343 lower lobe | Binary | 0.116 | 0.000 |
| Primary site: C348 overlapping lesion | Binary | 0.001 | 0.000 |
| Primary site: C349 lung, NOS | Binary | 0.000 | 0.000 |

Note: The table contains 58 baseline model terms plus the propensity-score distance as a diagnostic row. The propensity-score distance is not a baseline covariate. All 58 baseline model terms had |SMD|<0.10 after overlap weighting. SMD, standardized mean difference.

Supplementary Table S2. Overlap-weight distribution and effective sample size in the primary 5-year cohort.

| Surgical group | Raw n | Sum of overlap weights | Effective sample size | ESS / raw n | Maximum weight |
| --- | --- | --- | --- | --- | --- |
| Lobectomy | 3,642 | 366.53 | 2642.65 | 72.6% | 0.460 |
| Segmentectomy | 430 | 366.53 | 425.82 | 99.0% | 0.992 |

Note: ESS=(Σw)²/Σw². The overlap estimand retained nearly the full segmentectomy information while downweighting lobectomy observations with poor treatment equipoise.

Supplementary Table S3. Regional lymph-node evaluation as procedural context in the 2010–2020 cohort.

Panel A. Summary of examined regional nodes

| Surgery | N | Nodes known, n (%) | Median (IQR) | 0 nodes | ≥6 nodes | ≥10 nodes |
| --- | --- | --- | --- | --- | --- | --- |
| Lobectomy | 4,619 | 4,339 (93.9%) | 9 (5–15) | 3.2% | 71.8% | 48.2% |
| Segmentectomy | 604 | 570 (94.4%) | 5 (2–11) | 14.9% | 48.6% | 30.4% |

Panel B. Event counts by nodal-evaluation category

| Surgery | Nodes examined | N | Lung cancer deaths | Other-cause deaths |
| --- | --- | --- | --- | --- |
| Lobectomy | 0 | 138 | 35 | 32 |
| Lobectomy | 1-5 | 1,087 | 213 | 288 |
| Lobectomy | 6+ | 3,114 | 446 | 745 |
| Lobectomy | Unknown | 280 | 45 | 88 |
| Segmentectomy | 0 | 85 | 22 | 40 |
| Segmentectomy | 1-5 | 208 | 47 | 60 |
| Segmentectomy | 6+ | 277 | 31 | 40 |
| Segmentectomy | Unknown | 34 | 5 | 14 |

Note: Regional-node evaluation was considered a procedure-related characteristic and was not included in the baseline propensity-score model. These data are descriptive and should not be interpreted as evidence that nodal evaluation mediates the treatment association.

Supplementary Table S4. Continuous age interaction and descriptive age-stratified 3-year absolute risk differences.

Panel A. Formal continuous age-interaction tests

| Outcome | df | Wald χ² | P value |
| --- | --- | --- | --- |
| Lung cancer death | 3 | 5.29 | 0.152 |
| Other-cause death | 3 | 0.76 | 0.859 |

Panel B. Descriptive age-stratified 36-month cumulative incidence and risk differences

| Age group | N | Lobectomy n | Segmentectomy n | Lung CIF: lob | Lung CIF: seg | Lung RD/100 | Other CIF: lob | Other CIF: seg | Other RD/100 |
| --- | --- | --- | --- | --- | --- | --- | --- | --- | --- |
| 70-74 | 2,682 | 2,404 | 278 | 4.38% | 5.07% | +0.69 | 5.72% | 4.12% | -1.60 |
| 75-79 | 1,746 | 1,549 | 197 | 6.86% | 7.98% | +1.11 | 8.11% | 5.30% | -2.81 |
| 80+ | 795 | 666 | 129 | 9.22% | 8.44% | -0.77 | 9.83% | 12.22% | +2.39 |

Note: Age-stratified estimates are descriptive because the global continuous age-interaction test was primary and showed no statistically significant treatment-effect modification (P=0.152 for lung cancer death; P=0.859 for other-cause death). RD=segmentectomy minus lobectomy; CIF, cumulative incidence function.

Supplementary Table S5. Additional sensitivity analyses of 3-year absolute risk differences.

| Sensitivity analysis | Lung CIF: lob | Lung CIF: seg | Lung RD/100 | Other CIF: lob | Other CIF: seg | Other RD/100 |
| --- | --- | --- | --- | --- | --- | --- |
| Age ≥75 years | 7.92% | 8.37% | +0.44 | 8.49% | 7.96% | -0.52 |
| Age ≥80 years | 9.35% | 8.44% | -0.91 | 9.69% | 12.28% | +2.59 |
| Adenocarcinoma or squamous-cell carcinoma only | 5.86% | 6.98% | +1.12 | 7.13% | 6.19% | -0.94 |
| Exclude survival <1 month | 5.84% | 6.49% | +0.65 | 6.96% | 5.74% | -1.23 |
| 3-month landmark to 36 months | 5.36% | 6.52% | +1.15 | 6.53% | 5.38% | -1.15 |

Note: These prespecified sensitivity analyses report point estimates of overlap-weighted cumulative incidence and absolute RD. The source sensitivity outputs did not include bootstrap confidence intervals and are therefore interpreted descriptively. The 3-month landmark conditions on survival to 3 months and is not a baseline treatment-effect estimate.

Supplementary Table S6. Proportional-hazards diagnostics and temporal validation analyses.

Panel A. Proportional-hazards diagnostics

| Cohort | Outcome | Time transform | χ² | df | P value |
| --- | --- | --- | --- | --- | --- |
| Primary 5-year cohort | Lung cancer death | KM | 0.527 | 1 | 0.468 |
| Primary 5-year cohort | Other-cause death | KM | 0.560 | 1 | 0.454 |
| Primary 5-year cohort | All-cause death | KM | 1.045 | 1 | 0.307 |
| Secondary 3-year cohort | Lung cancer death | KM | 0.906 | 1 | 0.341 |
| Secondary 3-year cohort | Other-cause death | KM | 0.601 | 1 | 0.438 |
| Secondary 3-year cohort | All-cause death | KM | 1.368 | 1 | 0.242 |
| Primary 5-year cohort | Lung cancer death | Identity | 0.669 | 1 | 0.413 |
| Primary 5-year cohort | Other-cause death | Identity | 0.962 | 1 | 0.327 |
| Primary 5-year cohort | All-cause death | Identity | 1.260 | 1 | 0.262 |

Panel B. Continuous time-varying cause-specific hazard ratios

| Outcome | Month | HR (95% CI), segmentectomy vs lobectomy |
| --- | --- | --- |
| Lung cancer death | 12 | 1.12 (0.78–1.61) |
| Lung cancer death | 24 | 1.38 (1.05–1.81) |
| Lung cancer death | 36 | 1.56 (1.17–2.08) |
| Lung cancer death | 48 | 1.70 (1.22–2.36) |
| Lung cancer death | 60 | 1.82 (1.25–2.64) |
| Other-cause death | 12 | 0.91 (0.64–1.29) |
| Other-cause death | 24 | 0.97 (0.74–1.28) |
| Other-cause death | 36 | 1.01 (0.76–1.36) |
| Other-cause death | 48 | 1.04 (0.75–1.45) |
| Other-cause death | 60 | 1.07 (0.74–1.54) |

Continuous segmentectomy × log(time) interaction

| Outcome | β | 95% CI | P value |
| --- | --- | --- | --- |
| Lung cancer death | 0.301 | -0.010 to 0.613 | 0.058 |
| Other-cause death | 0.099 | -0.190 to 0.389 | 0.501 |

Panel C. Piecewise cause-specific Cox analyses

| Analysis | Outcome | Interval | HR (95% CI) | P value | Events |
| --- | --- | --- | --- | --- | --- |
| 36-month cut | Lung cancer death | 0-36 | 1.15 (0.79–1.66) | 0.464 | 284 |
| 36-month cut | Lung cancer death | >36-60 | 1.71 (1.15–2.54) | 0.008 | 199 |
| 36-month cut | Other-cause death | 0-36 | 0.81 (0.54–1.22) | 0.313 | 299 |
| 36-month cut | Other-cause death | >36-60 | 1.16 (0.80–1.70) | 0.429 | 266 |
| 24-month cut | Lung cancer death | 0-24 | 1.16 (0.73–1.85) | 0.524 | 184 |
| 24-month cut | Lung cancer death | >24-60 | 1.49 (1.07–2.08) | 0.018 | 299 |
| 24-month cut | Other-cause death | 0-24 | 0.70 (0.41–1.20) | 0.201 | 197 |
| 24-month cut | Other-cause death | >24-60 | 1.13 (0.82–1.55) | 0.468 | 368 |

Panel D. Conditional landmark analyses

| Landmark | Outcome | Eligible N | Lobectomy n | Segmentectomy n | HR (95% CI) | Conditional RD/100 | P value |
| --- | --- | --- | --- | --- | --- | --- | --- |
| 36-month landmark | Lung cancer death | 3,454 | 3,087 | 367 | 1.71 (1.15–2.54) | +3.44 | 0.008 |
| 36-month landmark | Other-cause death | 3,454 | 3,087 | 367 | 1.16 (0.80–1.70) | +1.09 | 0.429 |
| 24-month landmark | Lung cancer death | 3,663 | 3,272 | 391 | 1.49 (1.07–2.08) | +3.56 | 0.018 |
| 24-month landmark | Other-cause death | 3,663 | 3,272 | 391 | 1.13 (0.82–1.55) | +1.08 | 0.468 |

Note: The Schoenfeld tests did not reject proportional hazards. The lung cancer segmentectomy-by-log(time) interaction was borderline but not statistically significant at the conventional 0.05 threshold (P=0.058). Piecewise and landmark analyses should therefore be interpreted as supportive sensitivity analyses of temporal strengthening rather than definitive evidence of a time-dependent causal effect. Landmark analyses condition on remaining alive and event-free at the landmark.

Supplementary Figure S1. Overlap-weighted cumulative incidence of other-cause mortality after segmentectomy versus lobectomy in the primary 5-year cohort.

Note: Curves show the overlap-weighted cumulative incidence of death from causes other than lung cancer during 60 months of follow-up. At 5 years, the cumulative incidence was 14.43% after lobectomy and 14.01% after segmentectomy. The absolute risk difference was −0.42 deaths per 100 patients (segmentectomy minus lobectomy; 95% percentile bootstrap CI, −3.81 to +3.35), providing no clear evidence of a competing-mortality reduction after segmentectomy.

Supplementary Figure S2. Propensity-score overlap before weighting in the primary 5-year cohort.

Note: Density curves show the estimated propensity score for segmentectomy according to the observed surgical procedure before overlap weighting. The distributions overlap substantially in the clinically relevant middle range, supporting estimation of the average treatment effect in the overlap population (ATO). Overlap weighting downweights observations with near-deterministic treatment assignment rather than targeting the full eligible population.

Supplementary Figure S3. Annual use of segmentectomy and lobectomy among eligible resections, 2010–2023.

Note: The proportion of eligible resections performed as segmentectomy increased from 9.1% in 2010 to 24.5% in 2023, while the proportion performed as lobectomy decreased correspondingly. This utilization analysis is descriptive and uses the full 2010–2023 eligible cohort (n=7,275).

Additional sensitivity analyses added during revision

E-value analysis. Sensitivity to unmeasured confounding was quantified for the primary lung cancer cause-specific HR using the E-value framework. Because the 5-year lung cancer cumulative incidence was not rare, the primary calculation used an HR-to-RR conversion appropriate for a non-rare outcome; the rare-outcome HR approximation was retained as a sensitivity calculation. E-values quantify the minimum strength of association that an unmeasured confounder would need to have with both treatment and outcome, conditional on measured covariates, to move the observed association to the null.

Supplementary Table S7. E-value sensitivity analysis for unmeasured confounding.

| Analysis | HR (95% CI) | RR equivalent | E-value, point | E-value, CI limit | Interpretation |
| --- | --- | --- | --- | --- | --- |
| Primary, common-outcome conversion | 1.31 (1.05-1.63) | 1.21 | 1.71 | 1.23 | Quantifies sensitivity; does not exclude residual confounding |
| Sensitivity, rare-outcome approximation | 1.31 (1.05-1.63) | 1.31 | 1.95 | 1.29 | Quantifies sensitivity; does not exclude residual confounding |

Note: The primary E-value calculation uses the hazard-ratio-to-risk-ratio conversion for a non-rare outcome. The confidence-limit E-value uses the bound closest to the null.

Supplementary Table S8. Procedural-adequacy-restricted sensitivity analyses according to regional lymph-node evaluation.

| Nodes examined | N (Seg/Lob) | Seg ESS | 5-y lung CIF Lob | 5-y lung CIF Seg | Lung RD/100 (95% CI) | Lung csHR (95% CI) | 5-y other CIF Lob | 5-y other CIF Seg | Other RD/100 (95% CI) |
| --- | --- | --- | --- | --- | --- | --- | --- | --- | --- |
| >=1 | 3,618 (330/3288) | 326.3 | 10.75% | 14.74% | +4.00 (+0.17 to +8.04) | 1.27 (0.99-1.64) | 14.21% | 11.30% | -2.92 (-6.92 to +0.77) |
| >=6 | 2,526 (168/2358) | 165.8 | 9.38% | 13.56% | +4.18 (-1.04 to +9.58) | 1.12 (0.76-1.67) | 13.86% | 8.21% | -5.64 (-9.92 to -0.88) |
| >=10 | 1,626 (98/1528) | 96.1 | 10.11% | 11.99% | +1.88 (-4.58 to +8.59) | 1.00 (0.61-1.65) | 14.22% | 7.51% | -6.71 (-12.14 to -0.07) |

Note: Each restricted cohort was reweighted using the original baseline propensity-score specification. Regional nodes examined was not entered into the propensity model because it is a procedure-related/post-exposure characteristic. RD is segmentectomy minus lobectomy. Confidence intervals for RDs are percentile intervals from 1,000 bootstrap replicates with propensity scores and overlap weights re-estimated in every replicate. These are procedural-restriction sensitivity analyses, not baseline causal effects.

Supplementary Table S9. Leading causes of other-cause mortality in the primary 5-year cohort.

| Cause category | Lobectomy n | Lobectomy weighted % | Segmentectomy n | Segmentectomy weighted % |
| --- | --- | --- | --- | --- |
| Cardiovascular disease | 280 | 26.8% | 43 | 30.2% |
| Unknown/unspecified | 244 | 23.7% | 24 | 17.3% |
| COPD/chronic respiratory disease | 153 | 14.0% | 31 | 22.5% |
| Other specified causes | 151 | 13.8% | 20 | 14.6% |
| Cerebrovascular disease | 65 | 5.7% | 5 | 3.8% |
| Alzheimer disease | 43 | 4.1% | 6 | 4.2% |
| Pneumonia/influenza | 33 | 3.1% | 4 | 2.9% |
| Renal disease | 25 | 2.7% | 2 | 1.3% |
| Diabetes mellitus | 24 | 1.9% | 2 | 1.6% |
| Accidents/adverse effects | 31 | 3.2% | 0 | 0.0% |
| Sepsis | 13 | 1.0% | 2 | 1.5% |

Note: Percentages are overlap-weighted percentages conditional on other-cause death and are descriptive only. The original SEER COD to site recode labels were retained in the audit dataset before grouping. Categories are not intended to estimate causal effects of surgical extent on individual non-cancer causes of death.
